# A Comparison of Actigraphy and Sleep Diaries for Infants’ Sleep Behavior

**DOI:** 10.3389/fpsyt.2015.00019

**Published:** 2015-02-12

**Authors:** Wendy A. Hall, Sarah Liva, Melissa Moynihan, Roy Saunders

**Affiliations:** ^1^School of Nursing, University of British Columbia, Vancouver, BC, Canada; ^2^University of British Columbia Faculty of Medicine, Vancouver, BC, Canada

**Keywords:** infant, sleep problems, actigraphy, sleep diaries, behavioral symptoms

## Abstract

Detecting the effectiveness of behavioral interventions to reduce infant night-waking requires valid sleep measures. Although viewed as an objective measure, actigraphy has overestimated night-waking. Sleep diaries are criticized for only documenting night-waking with infant crying. To support potential outcome measure validity, we examined differences between sleep diaries and actigraphy in detecting night-waking and sleep duration. We recruited 5.5 to 8-month-old infants for a behavioral sleep intervention trial conducted from 2009 to 2011. Intervention (sleep education and support) and control groups (safety education and support) collected infant diary and actigraphy data for 5 days. We compared night-time sleep actigraphy with diary data at baseline (194 cases), and 6 weeks (166 cases) and 24 weeks post-education (118 cases). We hypothesized numbers of wakes and wakes of ≥20 min would be higher and longest sleep time and total sleep time shorter by actigraphy compared with diaries. Using paired *t*-tests, there were significantly more actigraphy night wakes than diary wakes at baseline (*t* = 29.14, *df* = 193, *p* < 0.001), 6 weeks (*t* = 23.99, *df* = 165, *p* < 0.001), and 24 weeks (*t* = 22.01, *df* = 117, *p* < 0.001); and significantly more night wakes of ≥20 min by actigraphy than diary at baseline (*t* = 5.03, *df* = 183, *p* < 0.001), and 24 weeks (*t* = 2.19, *df* = 107, *p* < 0.05), but not 6 weeks (*t* = 1.37, *df* = 156, n.s.). Longest sleep duration was significantly higher by diary than actigraphy at baseline (*t* = 14.71, *df* = 186, *p* < 0.001), 6 weeks (*t* = 7.94, *df* = 158, *p* < 0.001), and 24 weeks (*t* = 17.18, *df* = 114, *p* < 0.001). Night sleep duration was significantly higher by diary than actigraphy at baseline (*t* = 9.46, *df* = 185, *p* < 0.001), 6 weeks (*t* = 13.34, *df* = 158, *p* < 0.001), and 24 weeks (*t* = 13.48, *df* = 114, *p* < 0.001). Discrepancies in actigraphy and diary data may indicate accurate actigraphy recording of movement but not sleep given active infant sleep and self-soothing.

## Introduction

Increased understanding about the implications of children’s night-waking for children’s and caregivers’ health (1) has raised questions about how to accurately capture children’s night-waking in community settings. Actigraphy has been the recommended approach for “objective measurement” of infants’ and children’s sleep in contrast with more “subjective measures,” such as parent-completed infant sleep diaries. The actigraph is a wrist-watch-like device that continuously records motion day and night. Using computerized scoring algorithms, raw activity scores are translated to sleep-wake scores (2). A computer interface translates the data into a picture (actigram) of daily sleep and wake episodes for analysis (3). Actigraphy is non-intrusive and can be used in the home (4). Sleep diaries are simple, inexpensive, and suitable for recording sleep in natural environments (5). In sleep diaries, parents record standardized aspects of children’s sleep patterns over 24 h, such as length of settling, interventions to settle, number and length of waking periods, interventions to deal with waking periods, and rising time for night sleep. Data about any adverse events (e.g., illness) are also documented (5).

Actigraphy and sleep diaries have been important in the context of capturing infant behavioral sleep problems. Frequent infant night-waking, with crying, beyond 6 months is a common problem identified by parents, with up to 20% of infant behavioral sleep problems persisting (6–8). Interventions provide parents with strategies to assist children to learn to self-soothe to sleep so that, following brief arousals during the night, they do not signal parents (cry) for assistance (9). It is night-waking with signaling and interruption of parents’ sleep that reduces parental sleep quality and increases parental fatigue (10). Parents intervening to assist infants to return to sleep can potentially prolong each wake (11) and contribute to sleep fragmentation. It is infants’ ability to consolidate sleep by having less interrupted sleep at night (night-waking) that is an important indicator of sleep quality in infancy and toddlerhood (1). Consequently, interventions are anticipated to enable infants, with parent-identified behavioral sleep problems, to have less fragmented night-time sleep and longer night-time sleep periods.

Recent work has suggested that actigraphy has high false negative rates; it detects wakefulness when a child is sleeping (12, 13). Sadeh (2) indicated that the validity of actigraphic sleep-wake algorithms has been called into question by the low specificity of actigraphy for wakes. Specificity refers to how accurately a tool identifies a problem; in the case of low specificity, more individuals are inaccurately identified as having the problem, in this case, wakes (3). Meltzer et al. (3) indicated that studies have uniformly reported low specificity for actigraphs, particularly for infants, and concluded that actigraphy has a limited ability to accurately detect wake after sleep onset among pediatric populations. Such claims have been supported in studies comparing videosomnography and polysomnography and actigraphy. For preschool-aged children, Sitnick et al. (13) reported that, using videosomnography as the standard, actigraphy demonstrated high sensitivity for sleep and very low specificity for wakes. When Insana et al. (14) compared sleep-wake scores based on actigraphy and polysomnography for 22 healthy toddlers, they found low specificity; actigraphy was unable to discriminate wake from sleep.

Few studies have compared sleep diary and actigraphy data for infants. In a sample of Israeli infants with sleep disturbances, Sadeh (5) found high correlations for sleep onset and sleep duration between actigraphy and diary data. He reported that night-waking demonstrated much lower correlations and there was a significant increase in discrepancies between actigraphic and diary data during the treatment period. For 52 Japanese infants, Asaka and Takada (15) found high correlations between diary and actigraphy data for sleep onset, offset, and duration but much lower correlations for night wakes and wakes after sleep onset. In a longitudinal study of 20 infants’ sleep over the first 12 months of life, So et al. (16) reported no difference between actigraphs and sleep diaries for total percentage of sleep or wake over 24 h but actigraphs scored less time asleep and more time awake at 2, 5, 9, and 11 months compared with sleep diaries.

Sadeh (2) suggested that the poor correspondence between actigraphy and subjective reports for sleep quality (e.g., wakes) could be attributed to inaccuracy of actigraphy or inaccuracy of subjective reports. Correlations can be problematic when validating different instruments for sleep measurement because a perfect correlation can be found between any two instruments, even with widely divergent measurement scales, as long as the measures increase at the same proportional rate (3). Price et al. (17) argued that prospective time-use diaries, including sleep onset, wake time, numbers of night wakes, and duration of night wakes, are valid and produce more precise and accurate information than summary recall, such as questionnaires. Müller et al. (18) contended that, based on their findings for 90 Swiss infants, paper and electronic diaries are valid and well-accepted methods for assessing infant sleep. A problem with parent diaries of infant sleep has been a tendency for parents to omit items from diaries over time (15, 19). Dayyat et al. (20) expressed concern about responder bias on sleep diaries when families are experiencing children’s sleep problems.

The literature has been limited by cross-sectional studies, a wide age range of children studied, small sample sizes, and a reliance on examining correlations between actigraphic and sleep diary data. Because sleep diaries offer data about infant signaling, we argue that they are important indicators of parental perceptions of improvement in infants’ night-time sleep. Interventionists are interested in improving parents’ assessments of infant sleep problems and determining infants’ abilities to self-soothe, as an indicator of self-regulation. The inability for actigraphy to identify signaling and the expense involved in relying on actigraphic data to determine efficacy of sleep interventions suggest it is important to compare actigraphic measures and sleep diary data to evaluate their utility for determining intervention outcomes for night-waking in infants.

### Purpose

We compared actigraphy data for night-time sleep matched with prospective sleep diaries at three time points (baseline, 6 weeks post-education, and 24 weeks post-education) for 6-to-8-month-old children, with parent-identified behavioral sleep problems. For each time point, we hypothesized that numbers of night wakes and long wakes of 20 min or more would be significantly higher in the actigraphy data compared with the sleep diary data. We also hypothesized that longest night sleep time and total night sleep time would be significantly shorter in the actigraphy data compared with the sleep diary data.

## Materials and Methods

The data for this analysis are taken from a pragmatic randomized controlled trial of an intervention for 6-to-8-month-old Canadian infants with behavioral sleep problems. The objective was to: determine if randomization to a group cognitive-behavioral intervention for infant sleep problems compared to randomization to a group cognitive-behavioral safety placebo reduced the proportions of parents reporting their children had a severe sleep problem (based on a four-point severity scale) or having their child wake less than an average of two times per night over five nights by actigraphy at 6 weeks post teaching session.

Infants were recruited if they: (1) had no identified health problems; (2) were between the corrected ages of 5.5 and 8 months (they were 6 months by the intervention point); and (3) met the American Academy of Sleep Medicine (21) clinical Classification of Sleep Onset Association disorder (waking two or more times per night at least five nights per week). Parents were eligible to participate if they: (1) were biological or adoptive; (2) read and spoke English; (3) had access to a telephone; (4) lived in the study catchment area, and (5) comprised a single or two parent family with both parents committing to the study. Infants were excluded from participation if they had: (1) organic causes of sleep disruption; (2) developmental disability; and/or (3) chronic neurological or respiratory conditions. Parental exclusion criteria were: (1) diagnosed depression; (2) diagnosed sleep problems; and (3) working permanent night shifts.

The trial involved baseline data collection prior to randomization to either a sleep intervention or a safety placebo group. Parents received a group education session on sleep (intervention) or safety (control) followed by 2 weeks of support phone calls offered twice a week. The primary follow-up for assessment of the intervention was 6 weeks post-education. After data collection, parents in the intervention (sleep) group received a booklet reproducing the infant safety intervention and parents in the control (safety) group received a booklet reproducing the sleep intervention. A secondary follow-up occurred at 24 weeks post-education session. All parents gave informed signed consent after the study was approved by the institutional (university) and community research ethics boards, certificate number H09-00757. The trial registration numbers are: ISRCTN, 42169337, url: http://isrctn.org/ NCT00877162, url: http://clinicaltrials.gov/.

### Subjects

At baseline, most infants were male and first-born (Table 1). Infants’ mean age was 6.7 months and the majority was breastfed. Most infants’ parents were living with a partner; their average time in relationship was 6.4 years. Infants were members of families with an average of 1.3 children. Parents’ mean years of formal education was 17.5 and their family incomes ranged from less than $30,000 CAD (4.9%) per year to more than $110,000 CAD (41.3%) per year. The majority of families self-identified as Canadian. Ten percent and 12.5% selected Chinese and South Asian ethnicity, respectively.

**Table 1:** **Number of participants and primary caregiver and infant demographic data by data collection period**.

	Data collection period		
	Baseline	6 weeks	24 weeks
Number of participants	194	166	118
**VARIABLE**			
**Infant characteristics**
Mean infant age in months (SD)	6.70 (0.93)	8.72 (1.13)	12.85 (1.06)
Infant gender (valid percent/number)
Male	54.7% (105)	57.6% (95)	57.6% (68)
Female	45.3% (87)	42.4% (70)	42.4% (50)
First-born (valid percent/number)	77.1% (148)	77.8% (128)	78% (92)
Breastfeeding (valid percent/number)	88.5% (170)	89.7% (148)	54.3% (63)
**Primary caregiver characteristics**			
Living with partner (valid percent/number)	99% (190)	98.2% (162)	96.6% (114)
Mean age in years (SD)	34.41 (4.52)	34.23 (4.31)	34.05 (3.67)
Mean number of years in relationship (SD)	6.41 (3.82)	6.39 (3.74)	6.60 (3.34)
Mean number of children (SD)	1.29 (0.58)	1.29 (0.60)	1.26 (0.53)
Mean years of education (SD)	17.52 (2.69)	17.56 (2.73)	17.56 (2.73)
Income (valid percent/number)			
$ 10,000–29,999	4.9% (9)	4.9% (8)	4.3% (5)
$ 30,000–59,999	14.1% (26)	14.6% (24)	17.1% (20)
$ 60,000–89,999	17.4% (32)	17.7% (29)	17.1% (20)
$ 90,000–109,999	22.3% (41)	19.5% (32)	18.8% (22)
more than $110,000	41.3% (76)	43.3% (71)	42.7% (50)
Ethnicity (valid percent/number)
South Asian	12.5% (24)	9.7% (16)	8.5% (10)
Canadian	50% (96)	50.9% (84)	60.2% (71)
Chinese	9.9% (19)	9.7% (16)	5.1% (6)
European	10.4% (20)	11.5% (19)	14.4% (17)
Other	17.2% (33)	18.2% (30)	11.9% (14)

### Data collection

Micro-mini motionlogger actigraphs™, on infants’ ankles, were used in zero crossing mode for scoring sleep with an amplifier setting of 18 and a measured epoch length as 1 min. Meltzer et al. (3) indicated 1 min is the most common epoch used for pediatric actigraphy studies. Actigraphs were worn for 5 days and nights. Reports of minimal days of recording necessary for useful data have been mixed, ranging from 5 to 7 days (22, 23). A review conducted by the American Academy of Sleep Medicine indicated that zero crossing mode ignores the amplitude of movement, does not register the acceleration of movements, and can count high frequency artifacts as considerable movement (19) but it has been the most common mode used with Ambulatory Monitoring devices (3). The data analysis software used in this study relies on using zero crossing mode [Action 2.4 software, (24)] to incorporate Sadeh’s algorithm for differentiating sleep and wakes in infants less than 1 year of age (25).

In a review of 228 pediatric sleep research studies (3), 56% of the projects used the Micro-mini-motionlogger™ actigraph from Ambulatory Monitoring Inc. (AMI) based in Ardsley, New York, USA (26). Sadeh’s algorithm has been used in 65% of 228 pediatric sleep research studies up to and including 2010 (3).

Sleep diaries for infants were completed by the primary caregiver over 24 h for 5 days. Parents who were most active in settling and responding to their infants recorded bed times, length of settling time, types of interventions to settle their infants, the number and length of waking periods, types of interventions used to deal with waking periods, rising time for night sleep, and rising and settling times for daytime naps. They also noted any adverse events (e.g., illness).

### Data analysis

For analysis, actigraphy and sleep diary data were averaged over five nights at baseline, 6 and 24 weeks post-education session. We only included families with 5 days of actigraphy and diary data. We used SPSS 22 to analyze the diary and actigraphy data. We matched cases of subjects who had both diary and actigraphy data. Therefore, we used student’s paired *t*-tests to compare number of night wakes, number of long wakes of 20 min or more, longest night sleep time, and total night sleep time between sleep diaries and actigraphy. We did not compare settling time/sleep latency for a number of reasons. These young children had sleep onset association disorder (active interventions by parents to fall asleep) rather than limit setting sleep disorder. The mean for sleep latency at baseline was 0.58 min and at 6 and 24 weeks was 0 min. At baseline, actigraphy minutes for settling ranged from 0 to 66 min. Parents also reported settling their infants after they specified sleep onset on written diaries, which supported 0 min of sleep latency on actigraphy because infants were already asleep when parents placed them in their cribs. We used *t*-tests because Meltzer et al. (3) indicated that correlations are not an effective way to validate different instruments for sleep measurement. Results were considered statistically significant at the level of *P* ≤ 0.05.

## Results

Based on complete data available, we had varying sample sizes at baseline (222 infants with actigraphy data and 229 infants with diary data), 6 weeks post-education (192 infants with actigraphy data and 212 infants with diary data), and 24 weeks post-education (153 infants with actigraphy data and 183 infants with diary data). Available diary data exceeded actigraphic data because of mechanical losses with actigraphy and refusal by some parents to apply actigraphs to infants’ ankles. We matched diary and actigraphy cases and excluded cases without both actigraphy and diary data and five days of data. We conducted our analysis on the remaining 194 cases at baseline, 166 cases at 6 weeks, and 118 cases at 24 weeks. Our procedures resulted in a loss of 12.6% cases at baseline, 13.5% cases at 6 weeks, and 22.9% of cases at 24 weeks. We excluded some diary data because missing night wake duration data prevented us from calculating numbers of night wakes of 20 min or more, longest night sleep, and total night sleep duration for some of the diaries.

Our comparisons with paired *t-*tests supported our hypotheses. Tables 2–5 provide the means and standard deviations at each time point for numbers of night wakes, night wakes of 20 min or more, longest night sleep duration, and total night sleep duration respectively.

**Table 2:** **Comparison of the number of infant night wakes between actigraphy and parental diary data**.

Collection period	Actigraphy	Diary	*t*-test
	Mean (SD)	Mean (SD)	
Baseline	9.81 (2.93)	3.88 (1.44)	*t* (193) = 29.14**
6 weeks	8.73 (3.52)	2.39 (1.41)	*t* (165) = 23.99**
24 weeks	7.47 (3.18)	1.49 (1.23)	*t* (117) = 22.01**

*SD, standard deviation*.

**p < 0.001.

**Table 3:** **Comparison of the number of infant night wakes of 20 min or more between actigraphy and parental diary data**.

Collection period	Actigraphy	Diary	*t*-test
	Mean (SD)	Mean (SD)	
Baseline	1.25 (0.77)	0.97 (0.61)	*t* (183) = 5.03**
6 weeks	0.7 (0.55)	0.64 (0.61)	*t* (156) = 1.37^+^
24 weeks	0.49 (0.46)	0.39 (0.46)	*t* (107) = 2.19*

SD, standard deviation; ^+^*p* > 0.05; *p < 0.05; **p < 0.001.

**Table 4:** **Comparison of the longest infant sleep duration (minutes) between actigraphy and parental diary data**.

Collection period	Actigraphy	Diary	*t*-test
	Mean (SD)	Mean (SD)	
Baseline	166.92 (50.52)	256.41 (87.56)	*t* (186) = 14.71**
6 weeks	198.33 (73.07)	368.84 (131.55)	*t* (158) = 7.94**
24 weeks	241.63 (89.83)	455.23 (134.14)	*t* (114) = 17.18**

*SD, standard deviation*.

**p < 0.001.

**Table 5:** **Comparison of the total infant night sleep duration (minutes) between actigraphy and parental diary data**.

Collection period	Actigraphy	Diary	*t*-test
	Mean (SD)	Mean (SD)	
Baseline	565.65 (52.58)	591.28 (57.11)	*t* (185) = 9.46**
6 weeks	580.17 (51.44)	620.64 (54.80)	*t* (158) = 13.34**
24 weeks	587.82 (47.15)	624.87 (51.89)	*t* (114) = 13.48**

*SD, standard deviation*.

**p < 0.001.

The number of night wakes by actigraphy were significantly higher than by diary at baseline (*t* = 29.14, *df* = 193, *p* < 0.001), 6 weeks (*t* = 23.99, *df* = 165, *p* < 0.001), and 24 weeks post-education (*t* = 22.01, *df* = 117, *p* < 0.001). The number of night wakes of 20 min or more were significantly higher by actigraphy than by diary data at baseline (*t* = 5.03, *df* = 183, *p* < 0.001), and 24 weeks (*t* = 2.19, *df* = 107, *p* < 0.05), but not at 6 weeks (*t* = 1.37, *df* = 156, *n.s*.). Longest sleep duration at night was significantly higher using diary data than actigraphy at baseline (*t* = 14.71, *df* = 186, *p* < 0.001), 6 weeks (*t* = 7.94, *df* = 158, *p* < 0.001), and 24 weeks post-education (*t* = 17.18, *df* = 114, *p* < 0.001). Total night sleep duration was significantly higher based on diary data than by actigraphy at baseline (*t* = 9.46, *df* = 185, *p* < 0.001), 6 weeks (*t* = 13.34, *df* = 158, *p* < 0.001), and 24 weeks (*t* = 13.48, *df* = 114, *p* < 0.001).

To determine whether group assignment influenced differences in actigraphy and diary data, we divided the pooled groups into the intervention and control groups at 6 and 24 weeks post-education session (see Table 6). We found that all of our findings remained the same as the pooled group comparisons except for one variable. For night-waking of 20 min or more at 6 weeks, the intervention group had significantly fewer night wakes by diary data than by actigraphy data (*t* = 2.44, *df* = 77, *p* < 0.05) but the control group had no significant difference in night wakes of 20 min or more (*t* = 0.44, *df* = 78, *n.s*.). At 24 weeks, for night-waking of 20 min or more, the control group had significantly fewer night wakes by diary data than by actigraphy data (*t* = 2.24, *df* = 57, *p* < 0.05) but the intervention group had no significant difference in night wakes of 20 min or more when comparing diary with actigraphy data (*t* = 0.60, *df* = 49, *n.s*.).

**Table 6:** **Comparison of the number of infant night wakes of 20 min or more between actigraphy and parental diary data by group**.

Collection period	Group	Actigraphy	Diary	*t*-test
		Mean (SD)	Mean (SD)	
6 weeks	Intervention	0.70 (0.55)	0.54 (0.50)	*t* (77) = 2.44*
	Control	0.71 (0.55)	0.74 (0.60)	*t* (78) = 0.44^+^
24 weeks	Intervention	0.43 (0.45)	0.46 (0.42)	*t* (49) = 0.60^+^
	Control	0.52 (0.50)	0.36 (0.48)	*t* (57) = 2.24*

SD, standard deviation; ^+^*p* > 0.05; *p < 0.05.

Over time, we found an increased discrepancy between actigraphy and diary data. At baseline, mean number of night wakes was approximately 2.5 times higher by actigraphy. At 6 weeks, the mean number of night wakes was 2.65 times higher by actigraphy. By 24 weeks post-education, the mean number of night wakes by actigraphy was five times higher than diary data. For night wakes of 20 min or more, the pattern was not as clear. Comparing actigraphy data with diary data, the mean wakes of 20 min or more were 1.25 times higher at baseline, 1.1 times higher at 6 weeks, and 1.25 times higher at 24 weeks.

For longest night sleep duration, mean longest sleep period increased from 1.5 times higher by diary than actigraphy data at baseline to 1.86 times higher at 6 weeks, and 1.88 times higher at 24 weeks. For total night sleep duration, at baseline, mean length of total night sleep duration was about 1.5 times higher by diary data than actigraphy data. By 6 weeks, mean length of total night sleep duration was about 1.75 times higher, and by 24 weeks it was 1.8 times higher.

## Discussion

Our hypotheses were supported by our analysis. Our finding that numbers of night wakes by actigraphy were significantly higher at each time point than diary wakes supports other authors’ contentions that, for infants, actigraphy consistently over-estimates night-waking compared with diary data (5, 15, 16). So et al. (16) reported that their healthy infants studied over 12 months demonstrated more time awake (1.4 times) at night by actigraphy data compared with sleep diary results. Using the detection of body movement to identify wakefulness, given infants’ movement in active sleep, contributes to the differing parameters measured by diary and actigraphy data.

As indicated in our results, from baseline to 24 weeks post-education, actigraphy wakes increased from 2.5 times higher to 5 times higher than diary data. At 6 weeks, when only one group had been exposed to the intervention, number of wakes was about 2.65 times higher by actigraphy. By 24 weeks post-education, when both groups had been exposed to the intervention, albeit through different approaches, the number of wakes by actigraphy had increased to five times higher than diary data. Asaka and Takada (15) reported their healthy infant group (28 children under 1 year of age) demonstrated 1.5 times more night-waking by actigraphy than by diary data. High correlations of sleep diaries with actigraphic data have been reported when children are signaling their parents (5, 27, 28). When Sadeh (5) studied infants with parent-identified sleep problems who were receiving treatment, he reported that actigraphy wake means were 1.2 to 2 times higher than diary night-waking means, with the discrepancy increasing following an intervention for sleep problems. Our results suggest that, as infants learned to self-soothe following brief night-time arousals, movement defined by actigraphy as a wake was not detected by parents because infants were not signaling with arousals and likely self-soothing to sleep.

Our study is the first to report comparisons of night wakes of 20 min or more between actigraphy and diary data. Night wakes of that duration fit with Morrell (29) research criteria for a behavioral sleep problem, associated with the Infant Sleep Questionnaire. Our results were more mixed on that variable because, although there were significant differences between actigraphy and diary data at baseline and 24 weeks, there was no significant difference between actigraphy and diary data at 6 weeks post-education. Parental signaling is a likely accompaniment when infants wake for 20 min or more, thus making discrepancies in parental reports of wakes by diary data and actigraphy less likely than when infants are self-soothing. When we divided the pooled groups into the intervention and control groups at 6 and 24 weeks post-education session, this was the only variable where we detected any differences in the groups when comparing actigraphy and diary data. The findings from dividing the groups suggest that measurement of this variable following each group’s exposure to the intervention (the intervention group at 6 weeks and the control group at about 10 weeks post-education session) detected an improvement by diary data which was not detected by actigraphy data. In relation to the group differences, wakes of 20 min or more appear to be a sensitive indicator of responses to recent exposure to interventions to promote self-soothing.

Because our study is the first to report a comparison of diary and actigraphy data for longest night sleep duration, we could locate no comparisons from the literature. The statistically significant differences between actigraphy and diary data at each time point (more minutes by diary) and increased discrepancy from baseline to 24 weeks (1.5 times to 1.88) suggests to us that infants were self-soothing after brief night-time arousals and, thus, consolidating their sleep by parental report more effectively after exposure to the behavioral sleep intervention. If claims that actigraphy has high false negative rates are correct, specifically detecting wakefulness when a child is sleeping (12, 13), it is unsurprising that parental reports of longest sleep time exceed actigraphic estimations.

In our results, there were significant differences in night-time sleep duration at all three time points. Moreover, similarly to the actigraphic mean night wakes, we observed an increased discrepancy between estimates of mean minutes of night-time sleep duration over time. In other words, the mean values moved from 1.5 to 1.8 times higher for diary reports than for actigraphy from baseline to 24 weeks post-education. Our findings support other studies of infants that have reported diary estimates of night-time sleep duration overestimate sleep time compared with actigraphy data (15, 16). Asaka and Takada (15) found significantly higher estimates of infants’ night-time sleep duration by diary data than by actigraphy data. So et al. (16) reported their infants’ actigraphy results underestimated sleep compared with diary data, at 2, 5, 9, and 11 months of age. In contrast, Müller et al. (18) reported that there were no significant differences between actigraphy and diary indications of percentage time asleep at night.

Because we were measuring night-waking, night-waking of 20 min or more, longest night sleep time, and night-time sleep duration over about a 6.5-month period, there were changes in infants’ sleep patterns that could be attributed to developmental shifts. Nonetheless, we are comparing diary data and actigraphy data in this study; any developmental changes would affect measurement techniques similarly and both groups of infants (intervention and control) equivalently.

Our study had a number of limitations. While the cases were matched at each time period, variation in completeness of diary and actigraphic data by data collection period precluded us from comparing the same cases across all time points. We did not report on sleep latency. However, we argue that the concept of sleep latency should be applied with circumspection to infants because it implies deliberate settling with the intention of trying to sleep, which, while realistic for adults and older children, is less so for infants where parents impose settling, and in some cases, after the onset of sleep. While our approach fit with the recommendation that extended monitoring (5 days or longer) reduces the inherent measurement errors in actigraphy and increases reliability (2), 7 days of data collection would have provided stronger actigraphy data. Unfortunately, collecting actigraphic data for longer would likely have resulted in a trade-off with parents having more difficulty sustaining diary entries over an entire week, as Sadeh (5) reported.

From a technical standpoint, Ancoli-Israel et al. (19) observation that zero crossing mode ignores the amplitude of movement, does not register the acceleration of movements, and can count high frequency artifacts as considerable movement requires further consideration. In the pilot study, conducted prior to the trial (27), we used actigraphs in low-PIM setting and an algorithm from the University of California, San Diego. The only significant difference between the University of California algorithm and the Sadeh-ZCT (25) algorithm was in the number of epochs and their weighting before and after the index epoch; the algorithm used weighting to rescore the index epoch as sleep or wake. For the pilot study, each record was manually scored, and the difference was removed. After the manual scoring, the automated statistical analysis was run for final results. The pilot sample size was small (*N* = 39) and data were only collected for 3 days; however, our means for actigraphy wakes were much lower (1.7–5.6) in the pilot study than in the study reported here (7.5–9.8). Some of the challenges with over-estimating wakes may lie in using zero crossing mode for the Ambulatory Monitoring devices.

In conclusion, our results suggest that sleep diary data have an important role to play in determining outcomes following behavioral sleep interventions. Although there is the potential for bias, as suggested by Dayyat et al. (20), it is parents’ cognitions about infant sleep that we are trying to influence and infant signaling underlies parents’ concerns about infant sleep. Diary data are likely capturing infants’ self-soothing to sleep following brief arousals at night whereas actigraphy data seem to be capturing infants’ movement as wakes. In prospective parental sleep diaries, parents are only noting wakes associated with infant signaling (crying); however, as Bernier et al. (1) argued, it is important to consider sleep parameters that represent meaningful differences (fragmented sleep and poor sleep quality) in infancy.

## Author Contributions

WH was the principal investigator for the study, designed the study, supervised all of the data collection, and wrote the first draft of the manuscript. SL conducted all of the statistical analysis for the manuscript and contributed critical revisions to the manuscript. MM exported the data into SPSS, created the necessary data sets to conduct the analysis, and contributed critical revisions to the manuscript. RS processed and statistically analyzed all of the actigraphy records and contributed critical revisions to the manuscript.

## Conflict of Interest Statement

The authors declare that the research was conducted in the absence of any commercial or financial relationships that could be construed as a potential conflict of interest.
